# Non-limit passive earth pressure against cantilever flexible retaining wall in foundation pit considering the displacement

**DOI:** 10.1371/journal.pone.0264690

**Published:** 2022-03-11

**Authors:** Weidong Hu, Xinnian Zhu, Tao Hu, Weiwei Wang, Guozhi Lin

**Affiliations:** College of Civil Engineering and Architecture, Hunan Institute of Science and Technology, Yueyang, China; University of Vigo, SPAIN

## Abstract

A series of model tests are carried out on flexible retaining walls such as cantilevered piles, continuous walls, and sheet pile walls in the foundation pit to study the deformation, failure surface, and earth pressure distribution of soils in a passive zone. The shape, displacement, and shear strain of slip failure surface of sand in a passive area are analyzed by Particle Image Velocimetry. The slip failure surface is a broken line, the upper end slides out from the top of the soil, and the lower end is close to the zero displacements of the retaining wall. With the increase of the flexural deformation and horizontal displacement of the wall, the shear strain of the soil increases, and the shear fracture zone in the upper part of the sliding surface is more prominent. Based on the broken line rupture surface in the test results, the passive area can be divided into two zones, the limit state zone and the non-limit state zone. Then the mechanical models are set up respectively. Considering soil displacement, the upper and lower soil layer’s internal friction angle and wall-soil interface friction angle mobilize differently. The relationship between mechanical parameters along the retaining wall and horizontal displacement is estimated. Finally, the earth pressure distribution is obtained by using the horizontal differential layer method. The calculation results of this paper are consistent with the existing research results and the model test results in terms of earth pressure distribution. With the increase of depth, the unit earth pressure increases in the limit state zone. Still, after entering the non-limit state zone, the unit earth pressure rises to a certain extent and decreases rapidly.

## Introduction

Passive earth pressure is s necessary for the design of retaining structures in the foundation pit. The magnitude and distribution of passive earth pressure determine the safety and stability of the retaining structure. Many scholars have adopted different theoretical methods to solve the passive earth pressure. Kötter [1] first proposed the limit equilibrium differential equation of granular materials based on slip lines, which laid a theoretical foundation for rigorous mathematical solutions. Sokolovskii [2] obtained the passive earth pressure coefficient and the limit equilibrium differential equation of granular materials using the slip line method, which can strictly meet the stress equilibrium conditions and strength conditions. However, it needs to solve the approximate solution by numerical method for complex engineering problems [3]. Chen [4] used the upper bound analysis method to solve the earth pressure. Based on the wall soil friction, the upper bound solutions for various failure mechanisms were given. Soubra [5], Soubra and Macuh [6] employed the limit analysis maneuver approach to solving the coefficient, assuming translational multi-block failure and rotational log spiral mechanism. The limit analysis method does not require accurate determination of stress distribution within rigid blocks in the failure mechanism, but it usually cannot present the true solution. The finite element method [7, 8] considers the relationship between wall displacement and soil coordinated deformation and can obtain a more realistic earth pressure, but the calculation accuracy depends on the selection of soil constitutive models and the actual parameters are difficult to determine. Kame et al. [9], Patki et al. [10], and Alqarawi et al. [11] solved the passive earth pressure coefficient based on the static limit equilibrium theory. The limit equilibrium method assumes that the slip surface is the plane or curved surface passing through the toe of the wall, and the slip surface and the back of the wall reach the limit equilibrium state. The failure angle of the ultimate slip surface can be obtained by using the extreme value principle.

The passive earth pressure calculated by classical earth pressure theory assumes that the retaining wall is rigid, and the limit state design method is adopted. It is assumed that the failure surface of the soil in a passive limit state is a plane passing through the toe of the wall. Coulomb [12] takes that the slip plane forms an included angle with the horizontal plane. When the back of the wall is smooth, the angle between the slip plane and the horizontal plane is π/4- φ/2. Rankine’s theory and Coulomb’s theory solve the earth pressure based on the limit equilibrium method, and the distribution of passive earth pressure cannot be obtained. They are assumed to be linear distribution in engineering applications, far from the actual earth pressure distribution.

The supporting structures such as row pile wall, continuous wall, and sheet pile wall in foundation pit are flexible retaining walls because the thickness of this kind of retaining wall is very small compared with the height, and the wall has obvious flexural deformation, which cannot meet the assumption of a rigid body. When the cantilever flexible retaining wall of the foundation pit deflects, it presents triangular horizontal displacement, and the top displacement of the wall is the largest. The horizontal displacement varies nonlinearly along the wall. The mode and magnitude of soil displacement in the passive zone are significantly different from that of the rigid retaining wall. Thus, the displacement of the retaining wall in foundation pit engineering needs to be strictly limited. Most of the soil in the passive area does not reach the passive limit state, and the lower soil is actually in a non-limit conditon. Therefore, the classical earth pressure theory is not suitable for calculating of passive earth pressure against the cantilever flexible retaining wall in the foundation pit.

A lot of experimental studies and numerical analysis [13–17] show that the earth pressure against the retaining wall varies with the mode and magnitude of the displacement of the wall. In a non-limit state, the displacement significantly affects the mobilizations of the internal friction angle and the wall-soil interface friction angle. The stress path and stress state under the non-limit condition are substantially different from those under the limit state so that the resistance of soil in the passive zone varies with the displacement. At present, the elastic fulcrum method is used to calculate the passive resistance in the design of retaining structures in the foundation pit, and only the linear relationship between retaining wall deformation and displacement is considered. The displacement of the upper soil is larger and reaches the passive limit state. At this time, the strength parameters and the wall-soil interface friction angle reach the maximum. The displacement of the lower soil is smaller and in a non-limit passive state. The strength parameters and the wall-soil interface friction angle mobilize partly, and its magnitude has a nonlinear relationship with the soil displacement.

Therefore, in this paper, Particle Image Velocimetry (PIV) and earth pressure tests are carried out to study the shape of the sliding surface in the failure state under the flexural deformation mode of the cantilever flexible retaining wall and the broken line sliding failure surface is established based on the test results. Considering the displacement, strength parameters such as the internal friction angle and the wall-soil interface friction angle mobilize differently. The intensity is reduced, and the functional relationship between displacement and strength parameters is proposed. The differential element layer [18] is used to set up the mechanical model to determine the shape of the slip surface and the distribution of passive earth pressure.

## Model test

### PIV test

To study the shape of the failure surface of passive cohesionless soil under the flexural displacement mode of flexible retaining wall in the foundation pit, model tests are carried out and analyzed by Particle Image Velocimetry [19–21].

**Fig 1:**
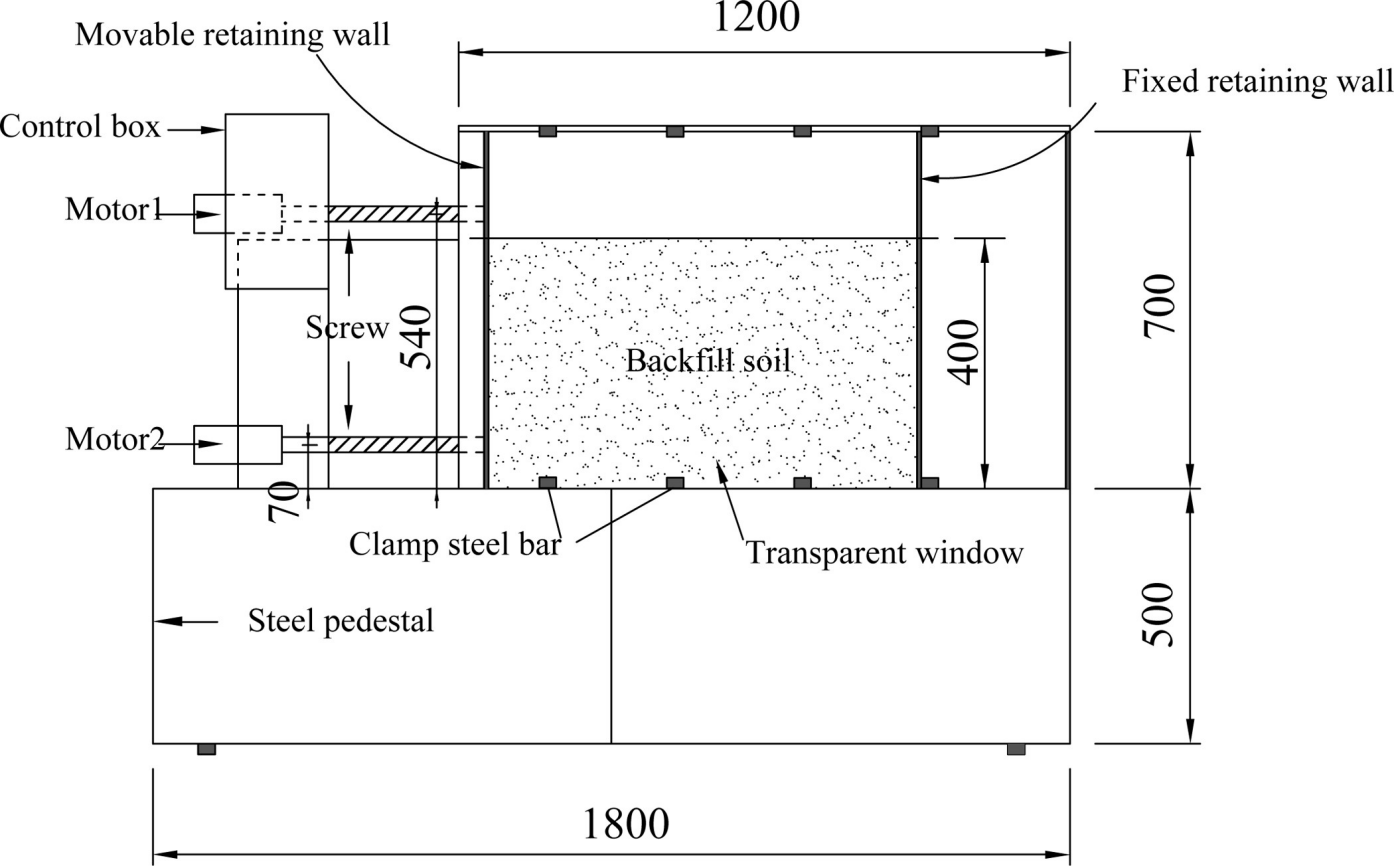
Test model box (unit: mm).

The passive earth pressure test of dry sand is carried out by using the self-made model. The size of the model is 1800mm long × 425mm wide × 1200mm high. As shown in Fig 1, the front side of the model box is coated with tempered glass, and the rear side is a steel plate. The left movable retaining wall is made of a 15mm thick polypropylene PP plate to simulate the structure in foundation pits such as sheet pile walls and diaphragm walls. The bending stiffness of the movable polypropylene wall is 154.13N·m^2^. Coarse sand is pasted on the surface of the PP plate with pitted double-sided adhesive to simulate the rough surface of the retaining wall. The fixed retaining wall on the right side is a 12mm thick steel plate.

**Fig 2:**
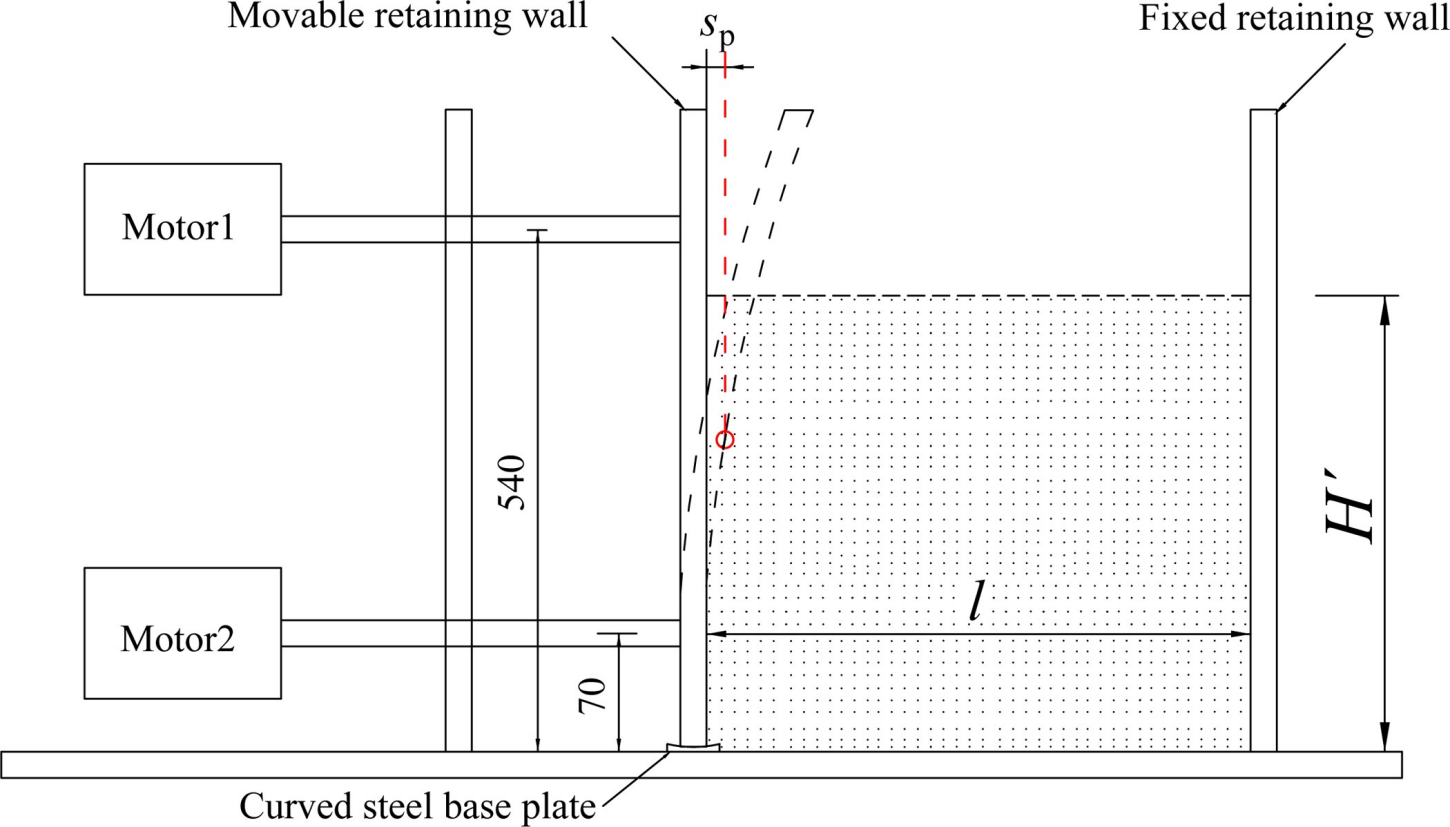
Deformation mode of flexible cantilever wall.

**Fig 3:**
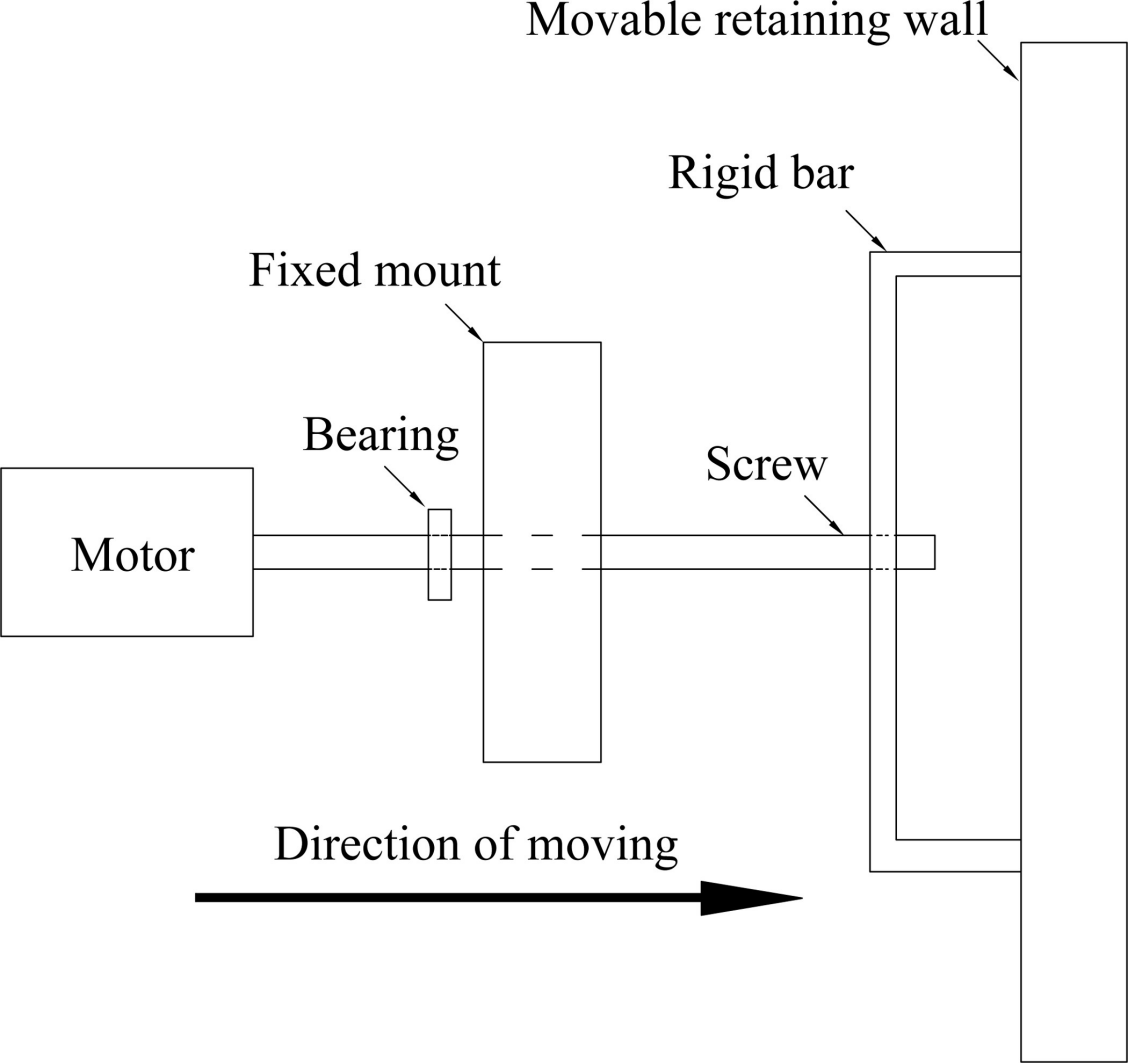
Motion control device of movable wall.

Figs 2 and 3 illustrate that the upper and lower motors are set outside the soil box to drive the screw to rotate. During the test, in order to simulate that the bottom of the movable retaining wall is embedded, the lower motor does not run. In order to prevent sand leakage at the bottom of the movable wall, a curved steel base plate is laid under the movable wall. Instead, the upper motor drives the upper moving shaft to rotate at a low speed so as to promote the movable retaining wall to move horizontally. The magnitude of displacement is measured by the dial indicator installed along the movable retaining wall. "*S*_P_" is assumed to be the horizontal displacement required for the passive soil to reach the limit state, when the external friction angle between the wall back and the soil in the pit reaches the maximum. The movable retaining wall squeezes the sand sample in the soil box. It moves along the potential sliding surface, which can simulate the sliding failure of the passive area in the foundation pit under the flexural deformation of the cantilever flexible retaining wall. PP plate has good resilience and can produce large elastic deformation. During horizontal loading, the retaining wall presents parabolic deformation to the inside of the foundation pit, which can better simulate the flexural displacement mode of flexible retaining walls such as cantilever sheet pile wall and diaphragm wall.

**Fig 4:**
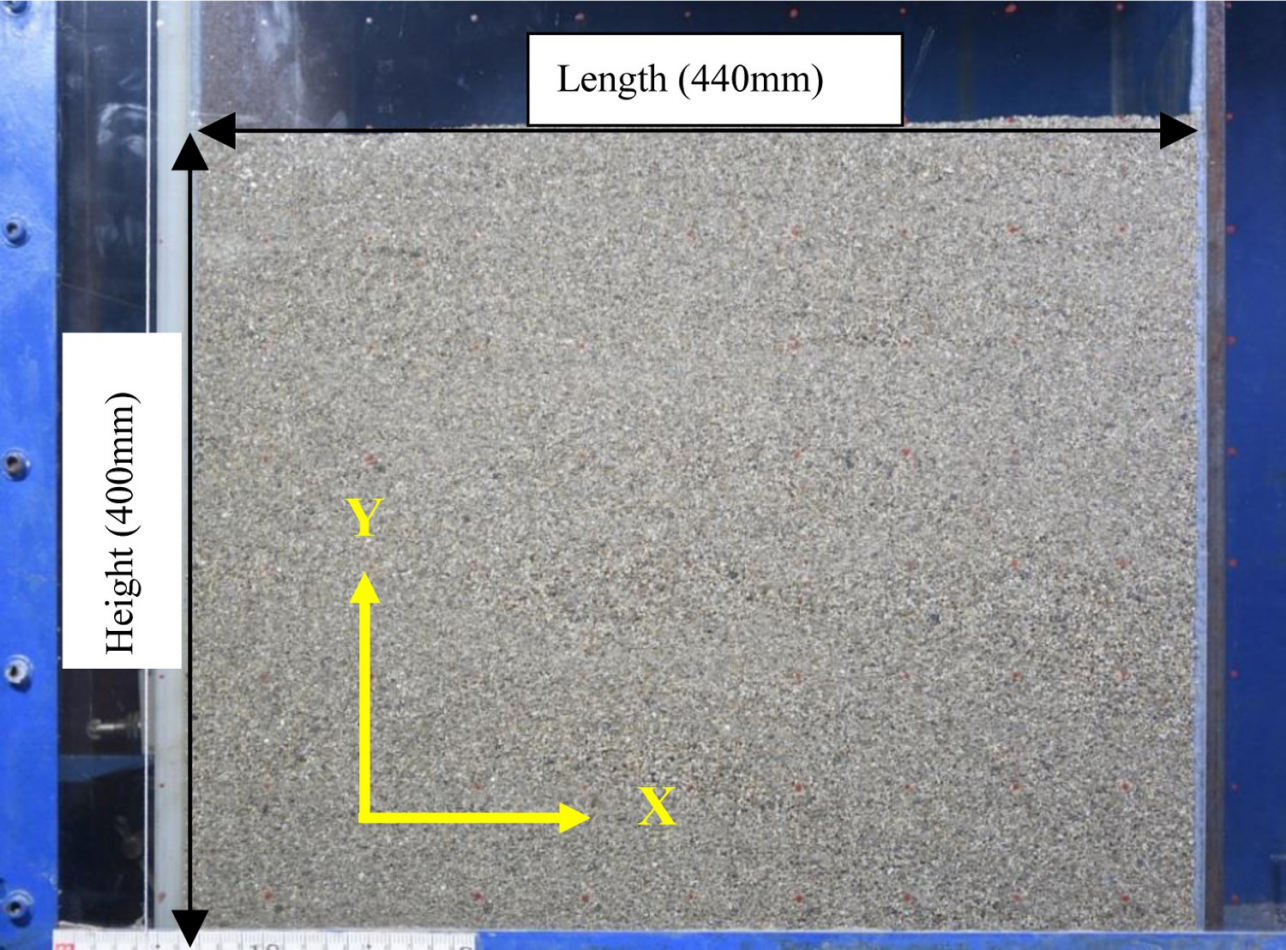
Sand sample in soil box.

The soil box is filled with 330/440mm length×400mm width×300/400mm height dry sand, used as the PIV analysis area, as shown in Fig 4. The digital camera is used to take pictures automatically, and the shooting time interval is 1s-2s. Finally, the image is processed by PIV analysis software. The soil sample for this model test is cohesionless sand, with a particle size of 0.075mm-0.63mm. In order to simulate the sand condition of the actual engineering, the natural falling sand method is used for sand filling in layers. A layer of sand is filled every 50mm, and then compacted in layers with the same standard. After filling, stand for more than 3 hours to eliminate the difference of layered compaction. The physical parameters of sand are shown in Table 1.

**Table 1:** Properties of the tested soil.

Unit weight(kN/m^3^)	Void ratio	Water content	Internal friction angle(°)	Wall-sand interface friction angle(°)
15.1	0.85	0	36.5	24.1

### Test results and analysis

**Fig 5:**
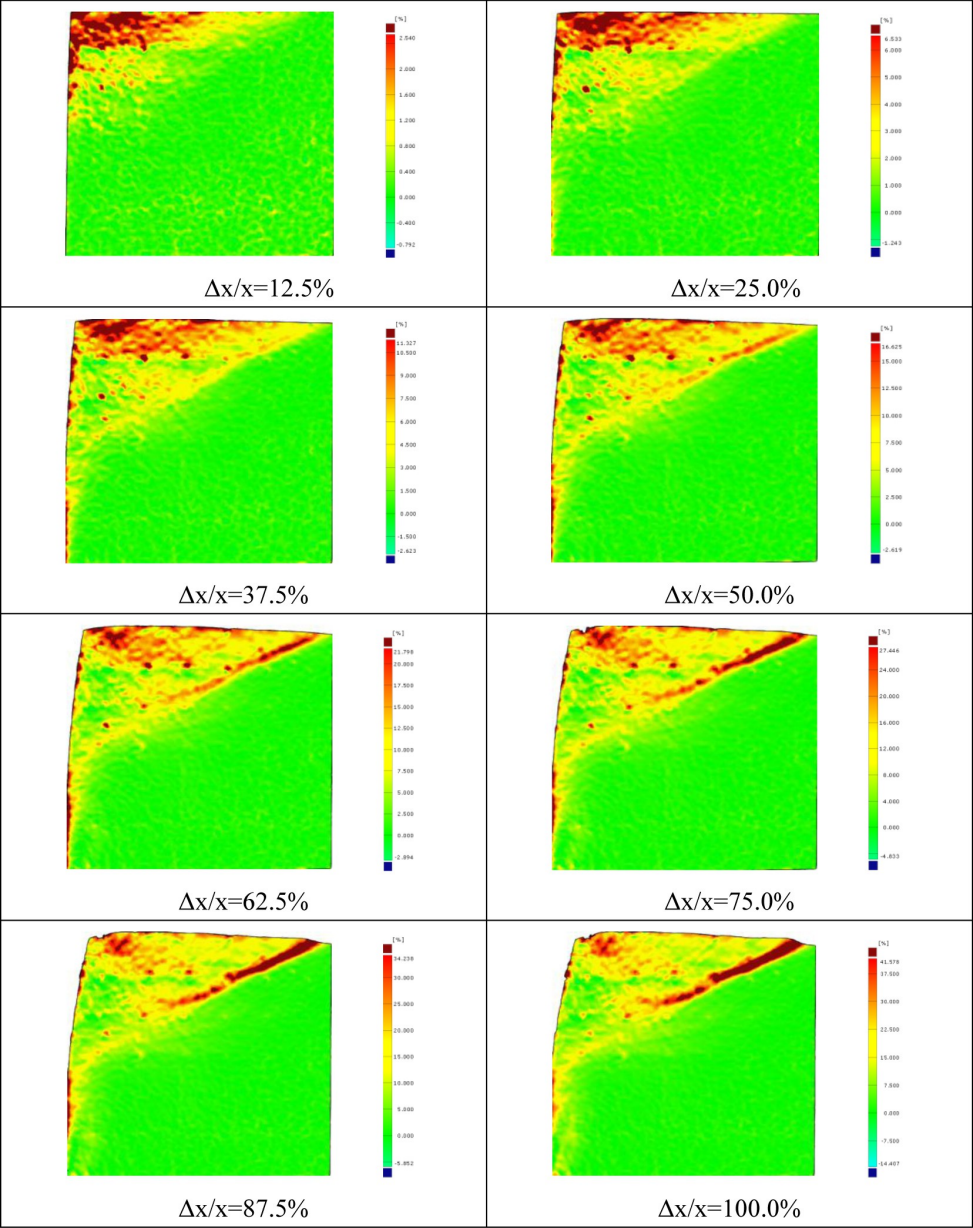
Shear strain nephogram of soil at different stages (300mm).

**Fig 6:**
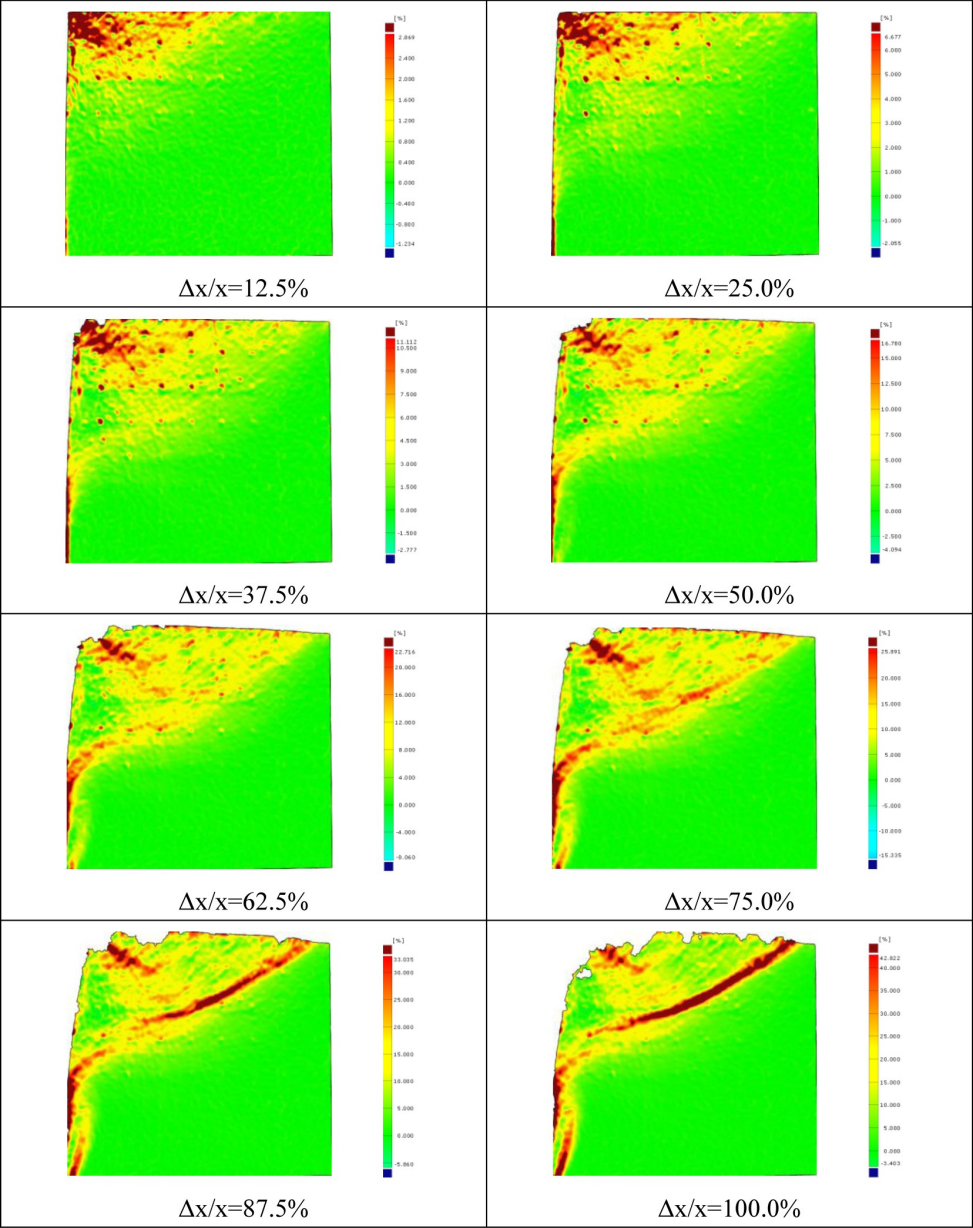
Shear strain nephogram of soil at different stages (400mm).

The continuous deformation and strain nephogram of soil under the flexural displacement mode of the cantilever flexible retaining wall is obtained using image analysis technology [20, 22]. Figs 5 and 6 show the soil strain nephogram of test sand with 300mm and 400mm filling height under different displacement states. The shear strain area is mainly concentrated at the top of the retaining wall cantilever in the initial stage. With the displacement increase the shear strain area gradually expands and develops downward, forming a sliding surface.

**Fig 7:**
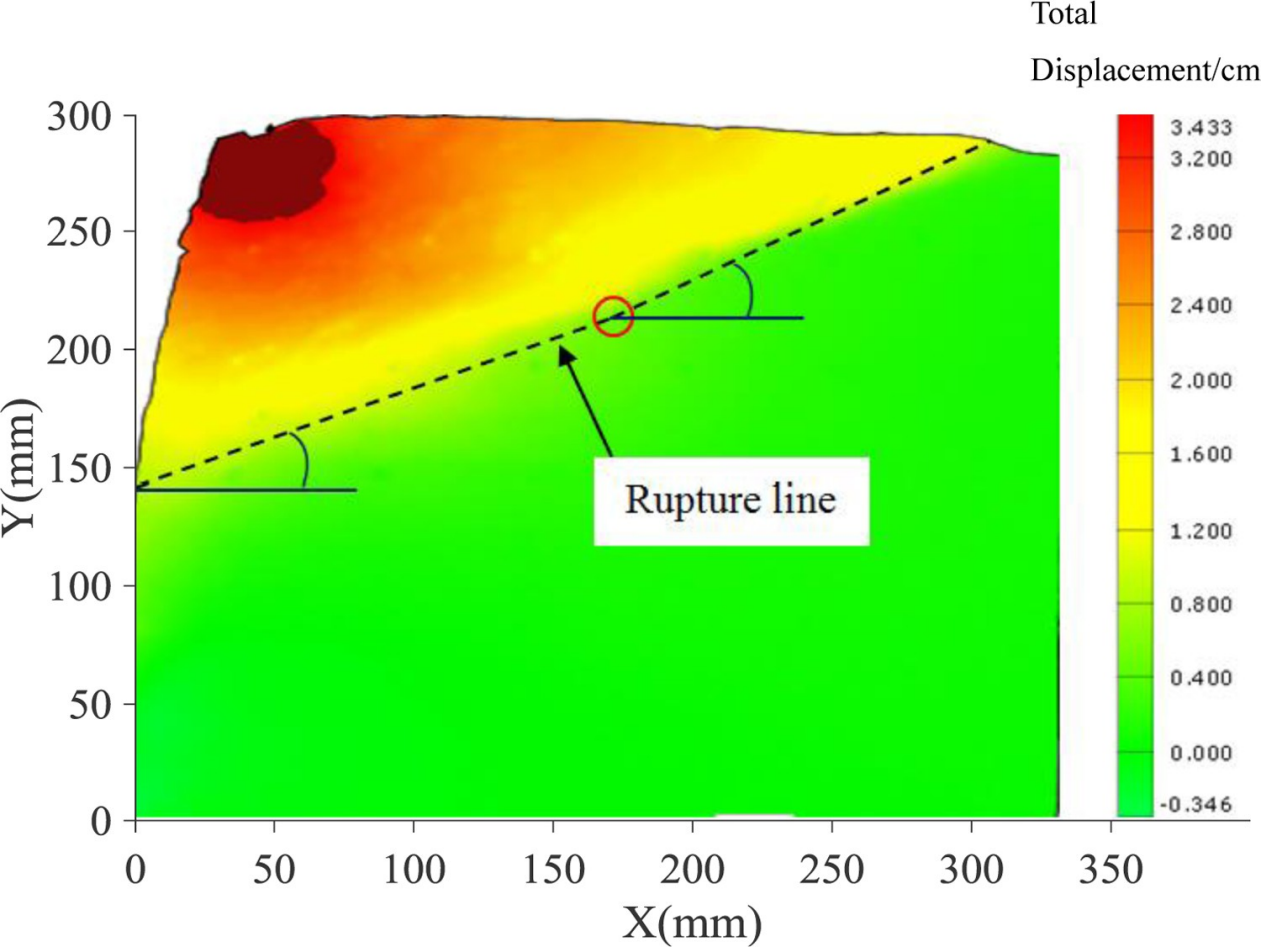
Nephogram of displacement in final state (300mm).

**Fig 8:**
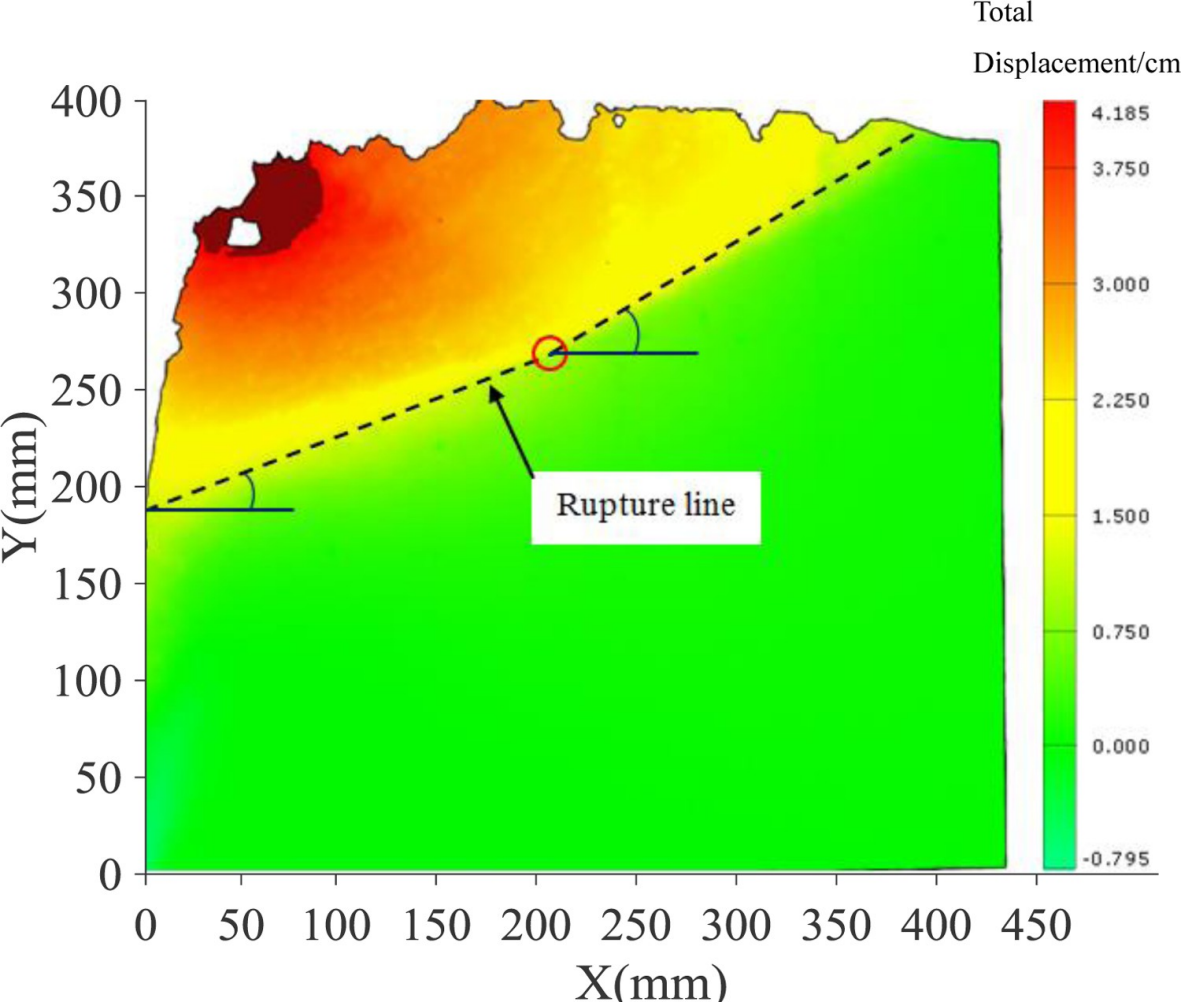
Nephogram of displacement in final state (400mm).

The displacement nephograms of the soil mass in the passive area reaching the final state are shown in Figs 7 and 8. The lower end of the sliding failure surface is close to the displacement zero-point passing through the middle of the wall body, and the upper end slides out of the top surface of the backfill. In the PIV test, it can be clearly observed that the sliding failure surface of the soil mass in the passive area is close to the broken line surface. The height of the starting point of the broken line slip surface obtained from the two groups of tests is different, and the height of the passive area in soil sample is also different. Fig 7 depicts that the height of passive area in 300mm high soil sample is 160mm (H / H ’ = 0.53), and the broken line surface is gentle. Fig 8 depicts that the height of passive area in 400mm high soil sample is 220mm (H / H ’ = 0.55), and the broken line surface is steep.

**Fig 9:**
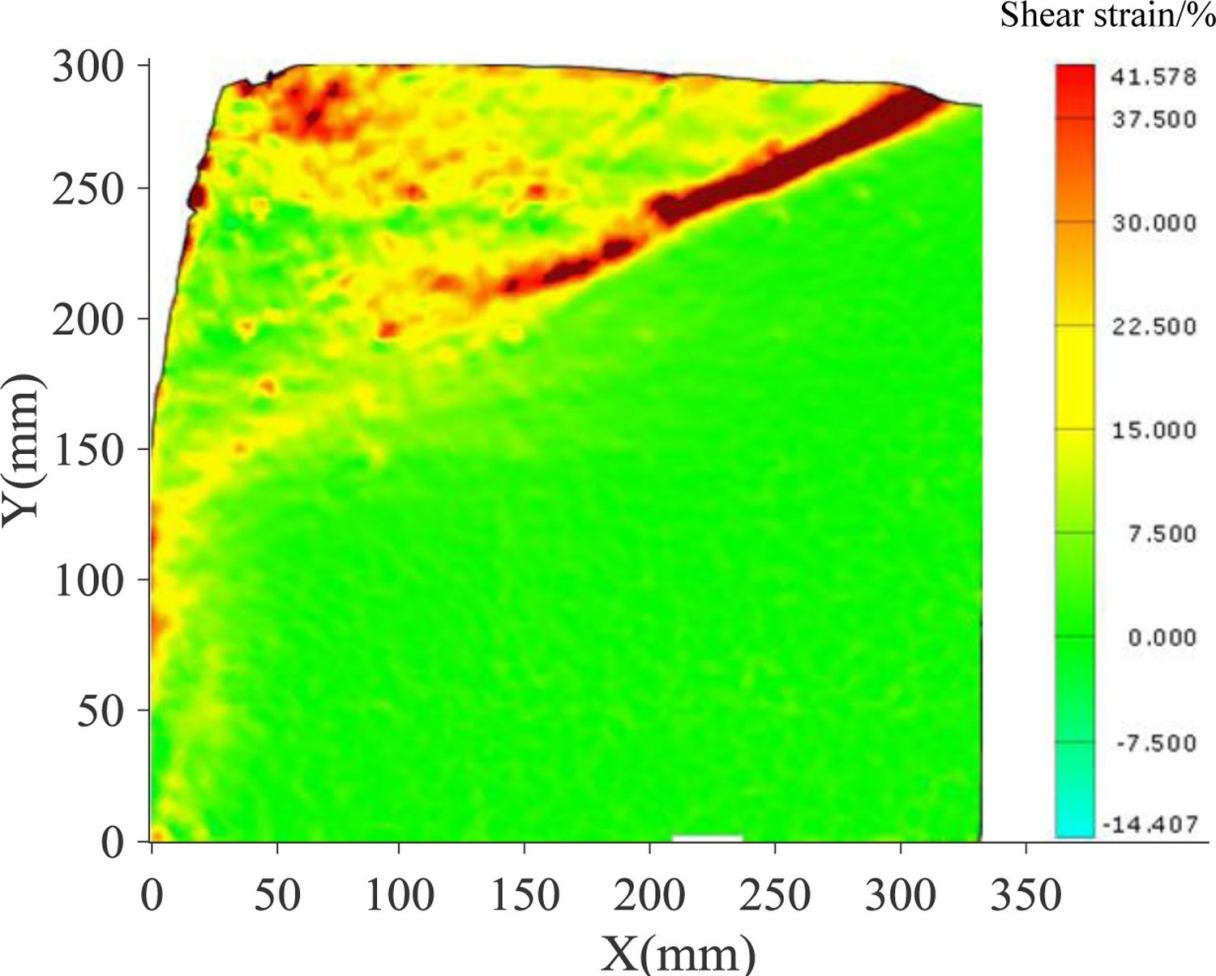
Nephogram of shear strain in final state (300mm).

**Fig 10:**
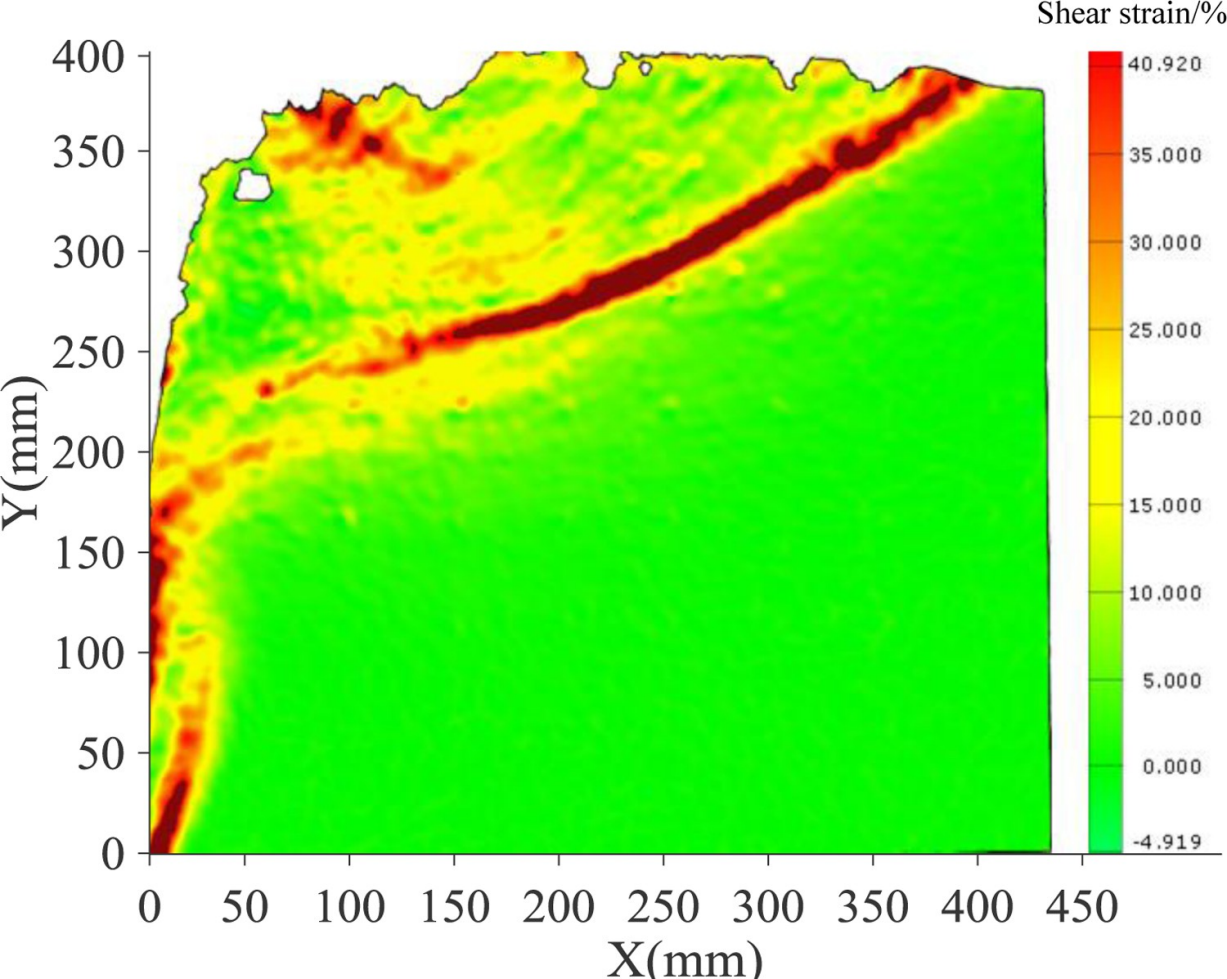
Nephogram of shear strain in final state (400mm).

Figs 9 and 10 illustrate the shear strain nephogram in the final failure state. It can be seen from the figure that a clear shear fracture zone is formed in the sliding failure area, and the shear strain at the upper part of the soil layer is more obvious. The fracture zone is a broken line fracture zone.

## Calculation for friction angle considering displacement

### Analysis model

The bottom of the foundation pit is horizontal, and the soil under the bottom of the foundation pit is cohesionless sand. Based on Coulomb theory, the potential slip surface of soil in passive area is a plane.As the flexible retaining wall flexes and moves towards the soil in the foundation pit, a potential sliding surface is gradually formed in the soil at the bottom of the foundation pit. When the horizontal displacement of the wall reaches the limit value, the soil in the foundation pit is destroyed along the sliding surface. Based on the previous test and analysis results, the bottom end of the sliding surface passes through the zero-point of horizontal displacement of the wall. The upper part develops obliquely upward and slides out of the top surface. The retaining wall is a flexible structure, its deformation is flexural, and the horizontal displacement along the retaining wall roughly presents a triangular distribution. The displacement zero-point appears at the lower part of the wall body. Its deformation mode is similar to the rotation of the wall body around the lower displacement zero-point.

**Fig 11:**
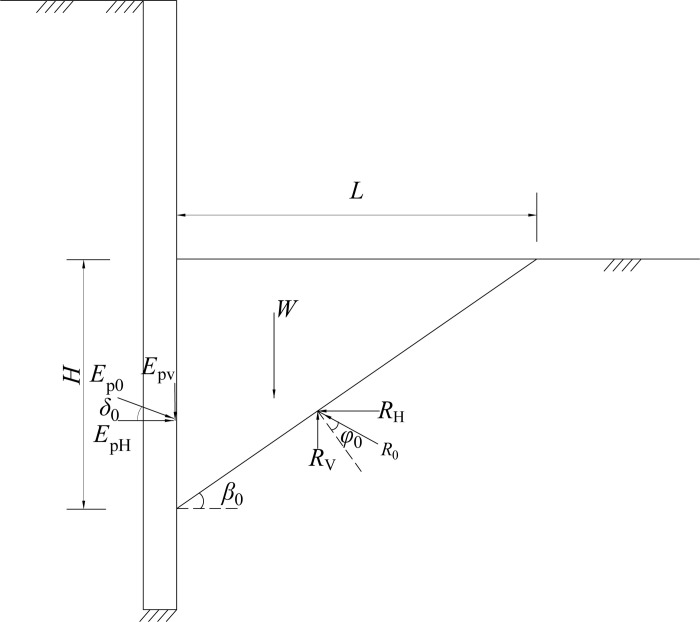
Stress mechanism of soil wedge (rest state).

**Fig 12:**
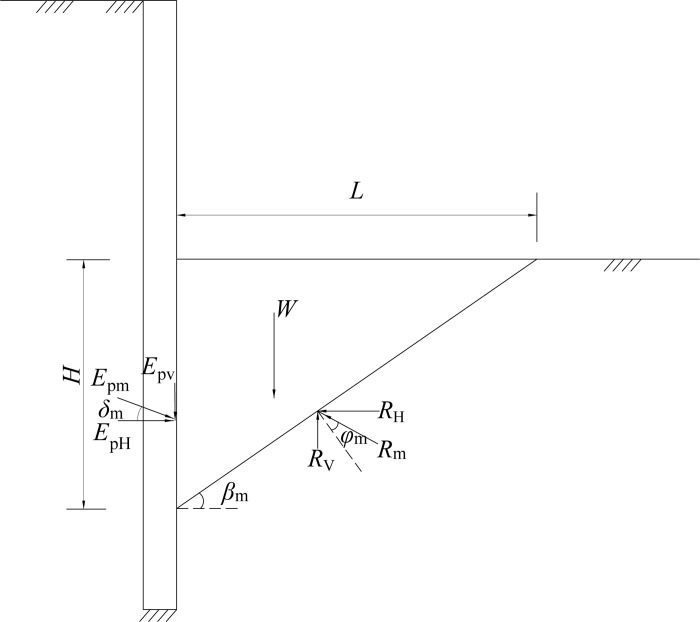
Stress mechanism of soil wedg (non-limit state).

**Fig 13:**
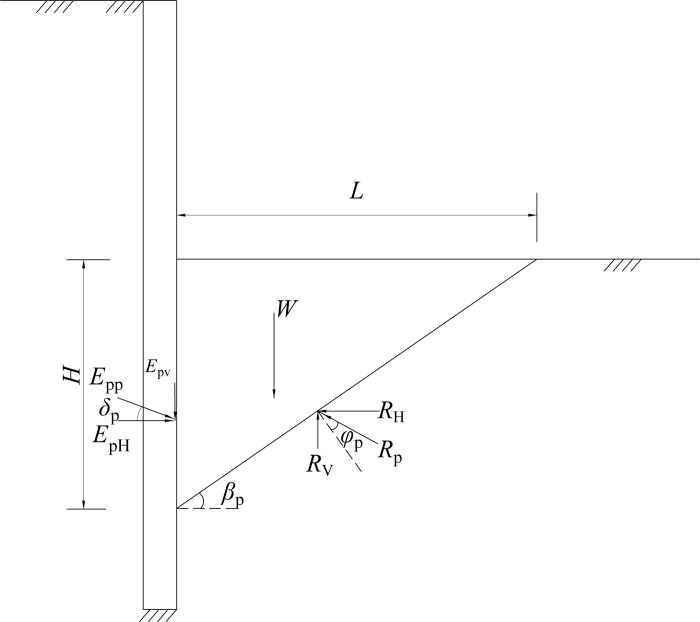
Stress mechanism of soil wedg (limit passive state).

Using model tests, Ichihara and Matsuzawa [23], Sherif et al. [24] have verified that the plastic sliding soil wedge is formed behind the wall. The passive area enters the limit state as the wall-soil interface friction angle reaches the maximum. Figs 11–13 illustrate the stress mechanism of the soil wedge as the flexible retaining wall moves towards the passive soil in the foundation pit. *H* is the height above the slip surface, that is, the height of failure soil. The required horizontal displacement of the wall is assumed to be *S*_P_, as the external friction angle between the wall back and the soil in the pit reaches the maximum. The actual internal friction angle of the soil and the wall-soil interface friction angle are expressed as *φ*_m_ and *δ*_m_, respectively. In Fig 11, when the horizontal displacement *S* = 0, the soil in the foundation pit is static, and the internal friction angle and the wall-soil interface friction angle are initial values. In Fig 13, when the horizontal displacement *S*>*S*_P_, the shear strength of soil reaches the limit, that is, the internal friction angle of the soil layer and the wall-soil interface friction angle get the maximum at the same time, and the unit earth pressure enters the passive limit. In Fig 12, when the horizontal displacement is 0<*S*<*S*_P_, the soil in the pit is in a non-limit state, and the internal friction angle of the soil is *φ*_m_, and the wall-soil interface friction angle *δ*_m_ are intermediate values.

**Fig 14:**
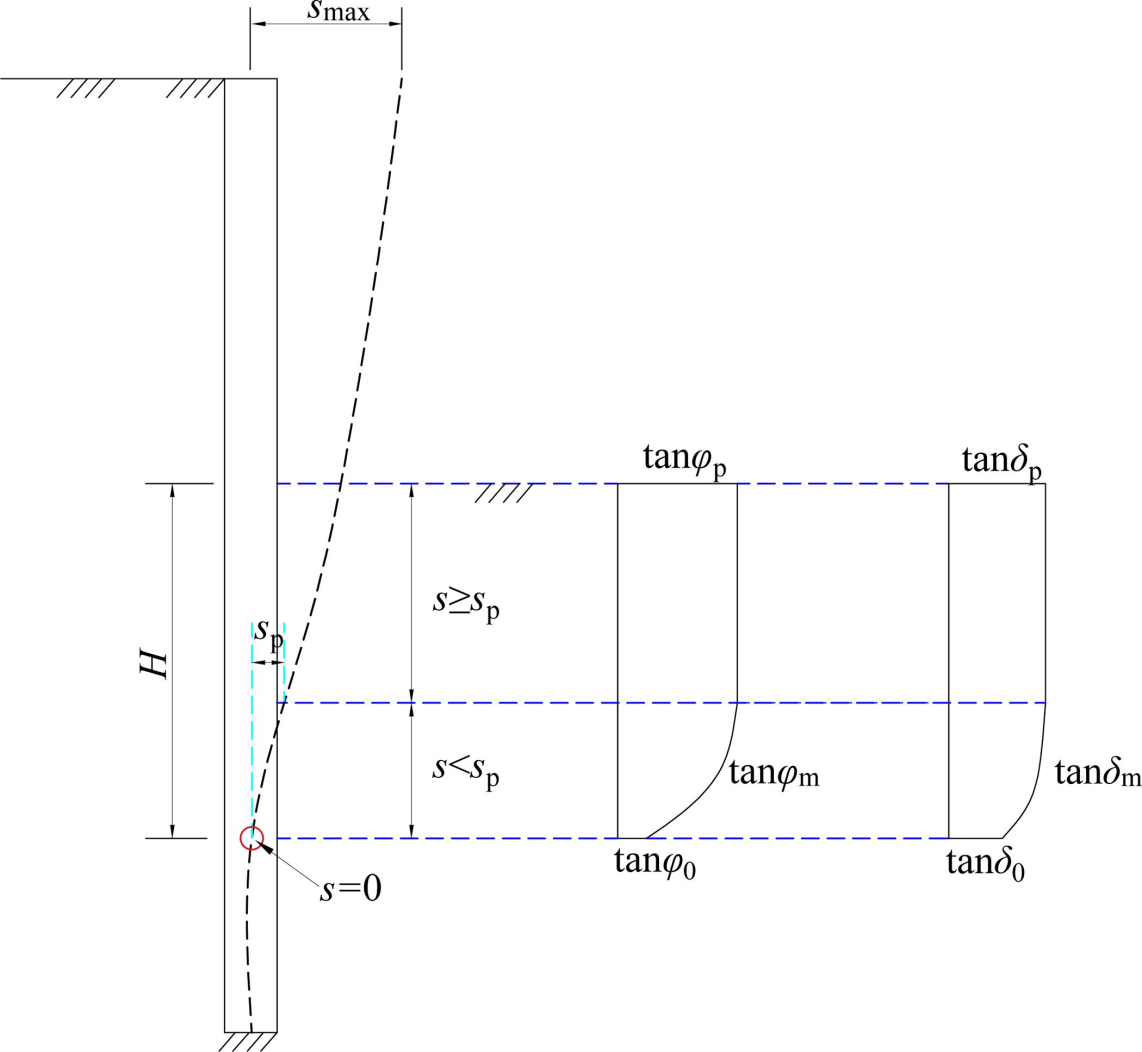
*φ*_m_ and *δ*_m_ varying along the height of retaining wall.

In the non-limit state, the internal friction angle of soil and the wall-soil interface friction angle is closely related to the horizontal displacement, which develops from the static state to the limit state. *φ*_m_ and *δ*_m_ increase accordingly as the horizontal displacement increases. The calculation formula for the variation of *φ*_m_ and *δ*_m_ (Fig 14) with the wall’s horizontal displacement can be expressed as

$$\left\{ \begin{array}{l} \tan\varphi_{m}=\tan\varphi_{0}+K_{d}(\tan\varphi -\tan\varphi_{0})\\ \tan\delta_{m}=\tan\delta_{0}+K_{d}(\tan\delta -\tan\delta_{0}) \end{array}\right.$$ (1)

Where *K*_d_ represents the effect of horizontal displacement on *φ*_m_ and *δ*_m_, *K*_*d*_ = 4 arctan(s/*s*_*p*_)/*π*

If the state is static, *S* = 0, then *K*_d_ = 0. At this time, the internal friction angle of the soil at the bottom of the pit and the wall-soil interface friction angle are *φ*_m_ = *φ*_0_, *δ*_m_ = *δ*_0_. In the limit state, *S* = *S*_P_, then *K*_d_ = 1. At this time, the internal friction angle and the wall-soil interface friction angle are *φ*_m_ = *φ*_p_ = *φ*, *δ*_m_ = *δ*_p_ = *δ*. The mechanical parameter *δ* can be determined by direct shear test. Matsuzawa and Hazarika [25] suggest the maximum value of the wall-soil interface friction angle *δ* = 2*φ*/3, which is applicable to the rough back of the wall. Considering that the back of the retaining wall is usually rough, “*δ* = 2*φ*/3” is recommended to parameter calculation, when the mechanical parameter cannot be obtained accurately [26]. For normally consolidated soil in a rest state, if the effect of the initial state on the wall-soil interface friction angle is not considered, then

$$\varphi_{0}=\arcsin(\frac{1-K_{0}}{1+K_{0}})$$ (2)

Meanwhile, *K*_0_ =1−sin *φ*, *δ*_0_ = *φ*/2

## Calculation for earth pressure in non-limit state considering displacement

When the retaining wall moves towards the bottom of the foundation pit, take the soil wedge under the non-limit state in Fig 12 as the isolation body, and assume that the included angle between the potential sliding surface and the horizontal plane is *β*_m_. The following equation can be obtained from the static equilibrium condition of the sliding wedge.


$$\{\begin{array}{l} E_{pV}+W=R_{m}\cos(\beta_{m}+\varphi_{m})\\ E_{pH}=R_{m}\sin(\beta_{m}+\varphi_{m}) \end{array}$$ (3)

In which, *E*_*pH*_ = tan *δ*_*m*_*E*_*pV*_,$W=\frac{1}{2}\gamma H^{2}\cot\beta_{m}$

$$E_{p}=\frac{1}{2}\gamma H^{2}\frac{\cos\beta_{m}\sin(\beta_{m}+\varphi_{m})}{\sin\beta_{m}\cos(\beta_{m}+\varphi_{m}+\delta_{m})}$$ (4)

H is the height of the potential sliding surface in the passive area at the bottom of the pit. Under a certain horizontal displacement state, *φ*_m_ and *δ*_m_ are specific values for the horizontal displacement *S*, and the *E*_p_ is only associated with *β*_m_. *E*_p_ has an extreme value as *β*_m_ vary so that the inclination *β*_m_ of the most dangerous potential slip surface can be obtained by using $\frac{dE_{p}}{d\beta_{m}}=0$

$$\beta_{m}=arc\cot[\tan(\delta_{m}+\varphi_{m})+\sqrt{\tan(\delta_{m}+\varphi_{m})[\cot\varphi_{m}+\tan(\delta_{m}+\varphi_{m})]}]$$ (5)

When the horizontal displacement S>S_P_, the inclination angle *β*_p_ of the slip surface is expressed as

$$\beta_{p}=arc\cot[\tan(\delta +\varphi )+\sqrt{\tan(\delta +\varphi )[\cot\varphi +\tan(\delta +\varphi )]}]$$ (6)

At rest, the inclination angle *β*_0_ of the potential slip surface is expressed as

$$\beta_{0}=arc\cot[\tan(\delta_{0}+\varphi_{0})+\sqrt{\tan(\delta_{0}+\varphi_{0})[\cot\varphi_{0}+\tan(\delta_{0}+\varphi_{0})]}]$$ (7)

Simplify the potential cracking surface shape

**Fig 15:**
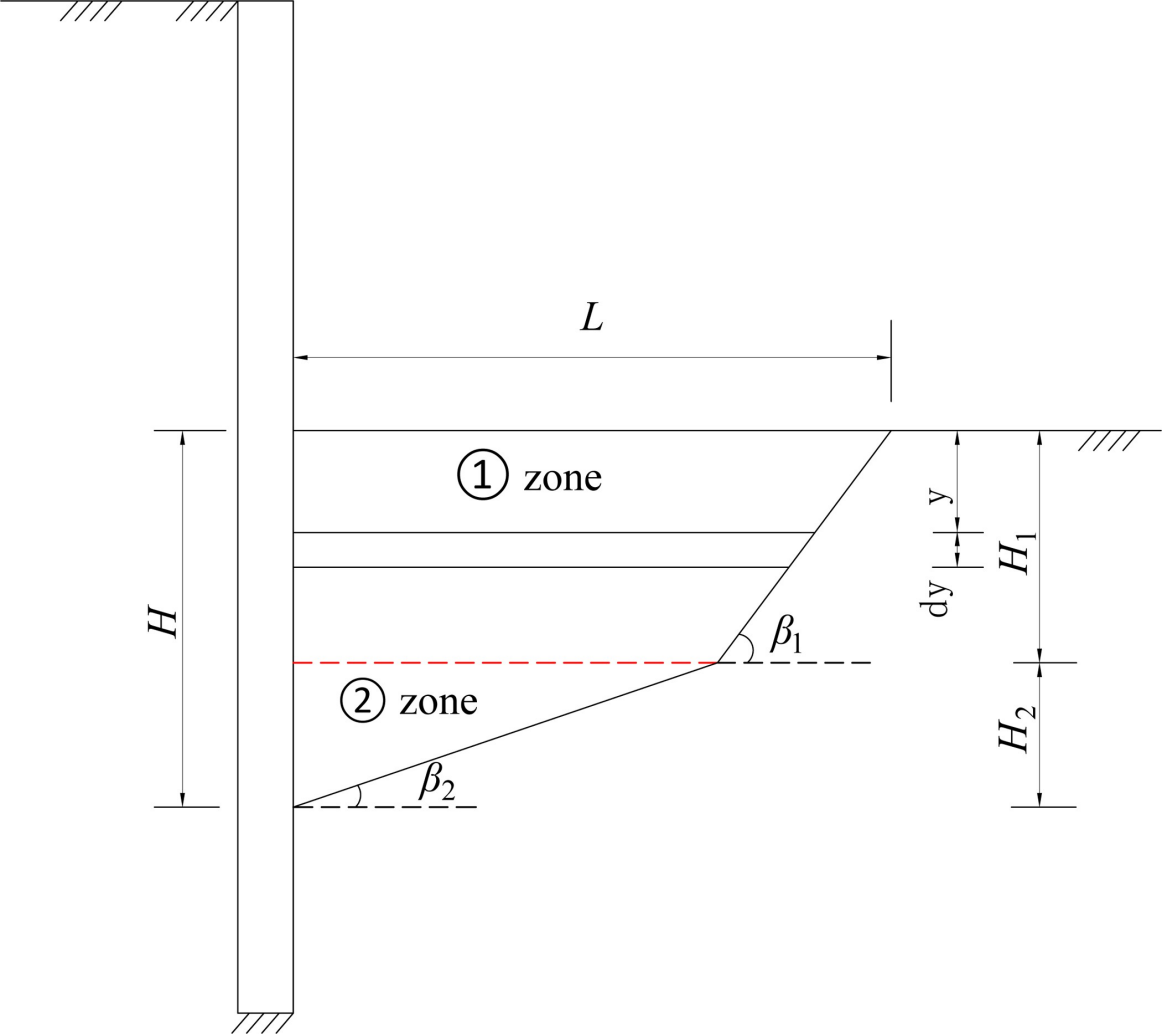
Sliding failure surface in passive area.

According to the previous introduction, the cantilever flexible retaining wall deflects and rotates around the displacement zero-point. The potential slip plane passing through the displacement zero-point appears in the passive zone at the bottom of the pit, as shown in the Fig 15. If the horizontal displacement of the upper soil is large and *S*>*S*_P_ within the height range of H_1_, the internal friction angle of the upper soil layer and the external friction angle of the wall soil reach the maximum, and the soil enters the passive limit state. There is an obvious sliding plane in the soil within the height of H_1_, and the included angle between the sliding plane and the horizontal plane is *β*_1_ = *π*/4-*φ*/2

If the horizontal displacement of the lower soil is small and *S*<*S*_P_ within the height of H_2_, the internal friction angle of the lower soil layer and the wall-soil interface friction angle are the intermediate value, which varies nonlinearly along with the height of the wall, and the soil is in the passive non-limit state. The potential slip surface within the height of H_2_ is a varying plane, and its inclination angle is between *β*_P_ and *β*_0_. Therefore, in order to simplify the calculation, the included angle can be defined as *β*_2_ = (*β*_0_+*β*_*p*_)/2. A broken line potential slip surface is formed in the passive area at the bottom of the foundation pit.

### The unit earth pressure

**Fig 16:**
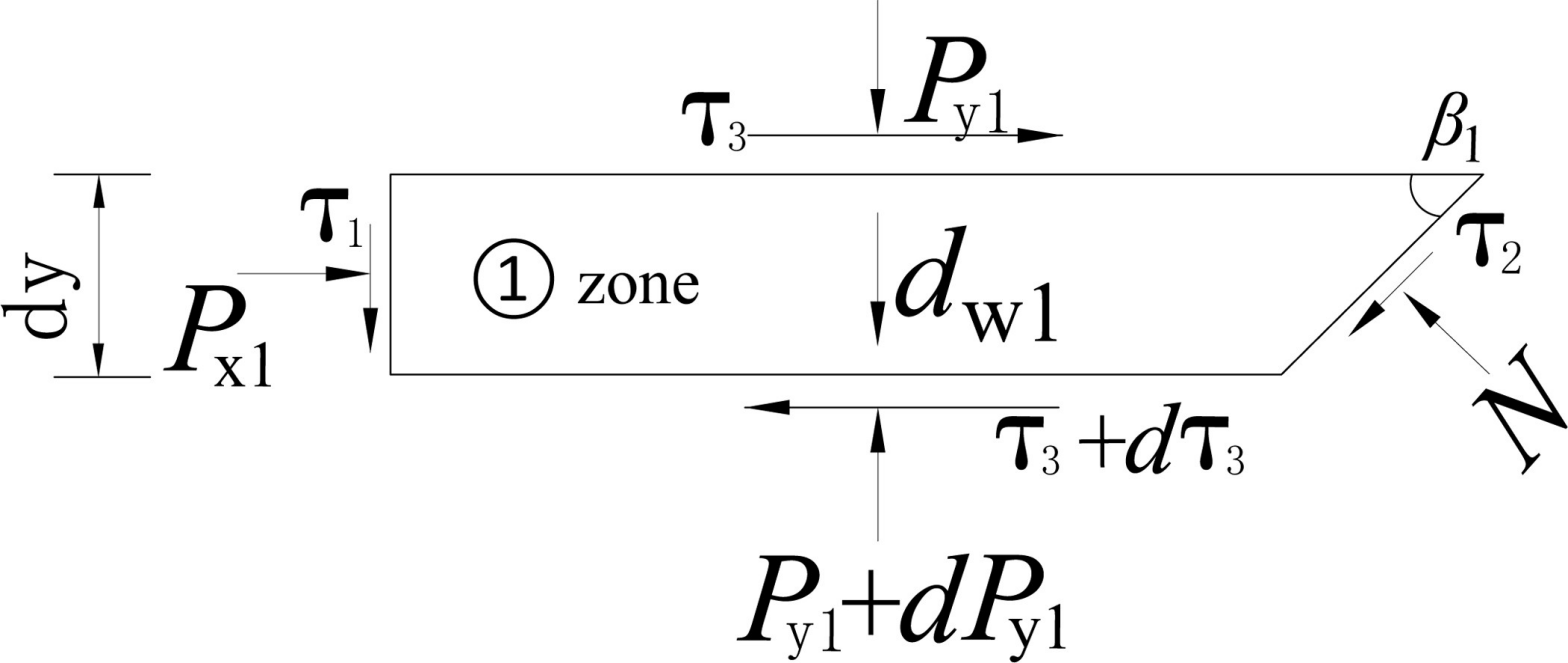
Forces analysis on zone ①.

The soil slides along the wall’s back and the broken plane, passing the displacement zero-point. The sliding soil wedge causes earth pressure. A horizontal differential element with a thickness of *d*y is taken from the sliding wedge with a depth of *y*, as shown in Fig 16.

The compressive stresses acting on the top and bottom surfaces of the unit are *P*_y1_ and *P*_y1_+d*P*_y1_, respectively, the lateral compressive stress on the retaining wall is *P*_x1_, and the shear stress on the wall soil interface is *τ*_1._ The shear stress on the tangent plane of the sliding surface is *τ*_2_. The normal stress perpendicular to the sliding surface is *N*, and the self-weight of the unit is *d*_w1_, as shown in Fig 16.

The static equation is obtained considering the horizontal static equilibrium condition.


$$P_{x1}+(\tau_{3}-\tau_{2})\cot\beta_{1}-N=\frac{d\tau_{3}}{dy}[H_{2}\cot\beta_{2}+(H_{1}-y)\cot\beta_{1}]$$ (8)

In which, *τ*_2_ = *N* tan *φ*, *τ*_3_ = *P*_*y*1_ tan *φ*. The solution process of *P*_x1_ is included in the S1 Appendix.

**Fig 17:**
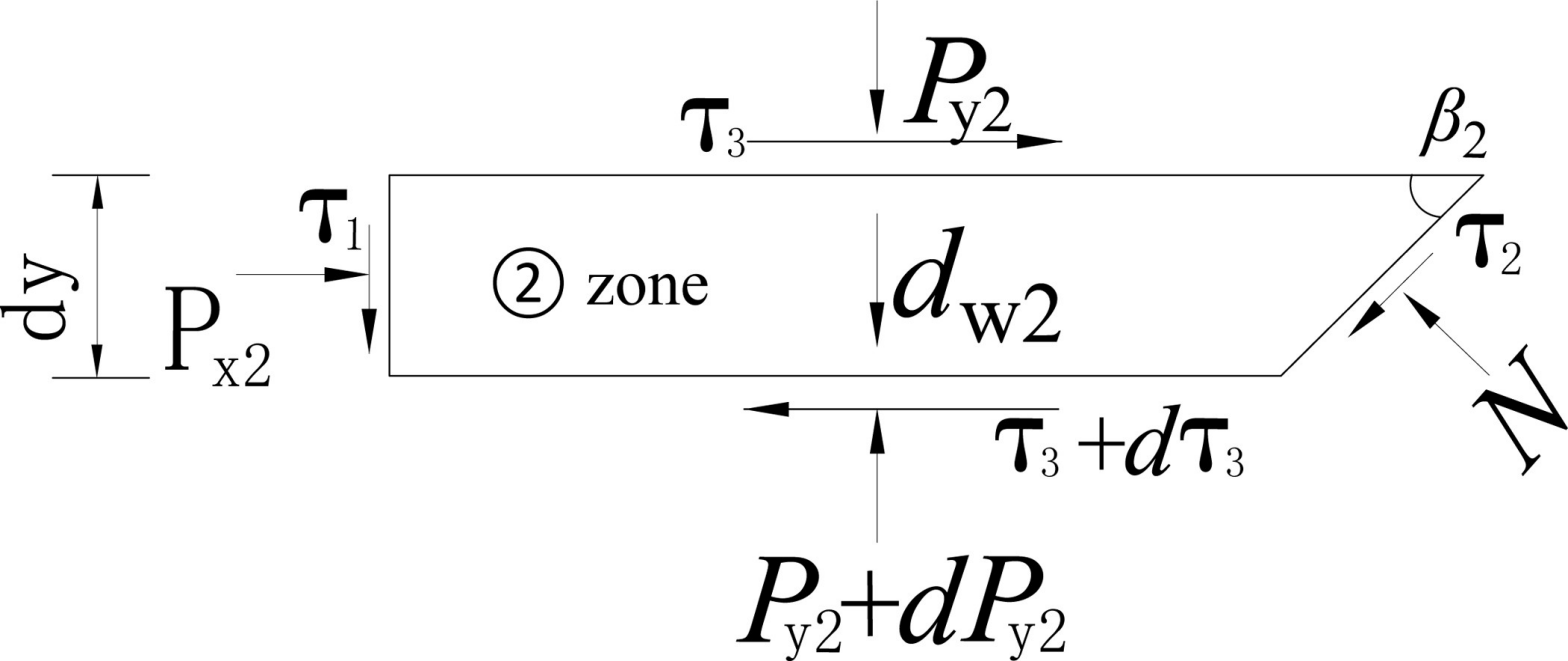
Forces analysis on zone ②.

In the same way, the zone ② differential unit is used as isolation, as shown in Fig 17. The horizontal equilibrium differential equation is presented

$$P_{x2}+(\tau_{3}-\tau_{2})\cot\beta_{2}-N=\frac{d\tau_{3}}{dy}(H-y)\cot\beta_{2}$$ (9)

The solution process of *P*_x2_ is included in the S2 Appendix.

The horizontal component *E*_px_ of the resultant force of passive earth pressure can be obtained by integrating *P*_x_ along the wall.


$$E_{\mathrm{px}}=\int_{0}^{H_{1}}P_{x1}d\theta +\int_{H_{1}}^{H}P_{x2}d\theta$$ (10)

### Analysis and comparison

#### Comparison with model test results

**Fig 18:**
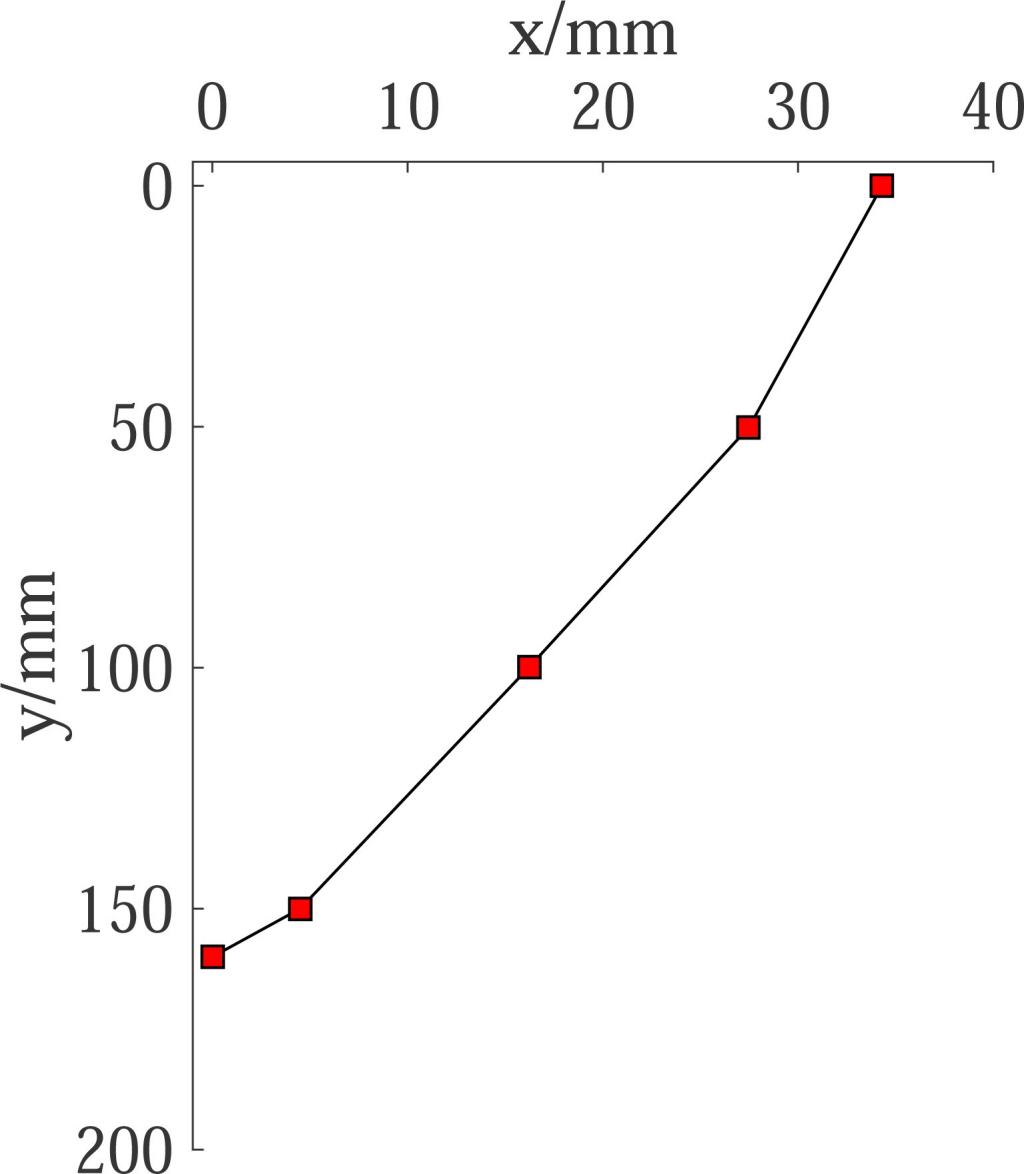
Displacement of cantilever retaining wall.

**Fig 19:**
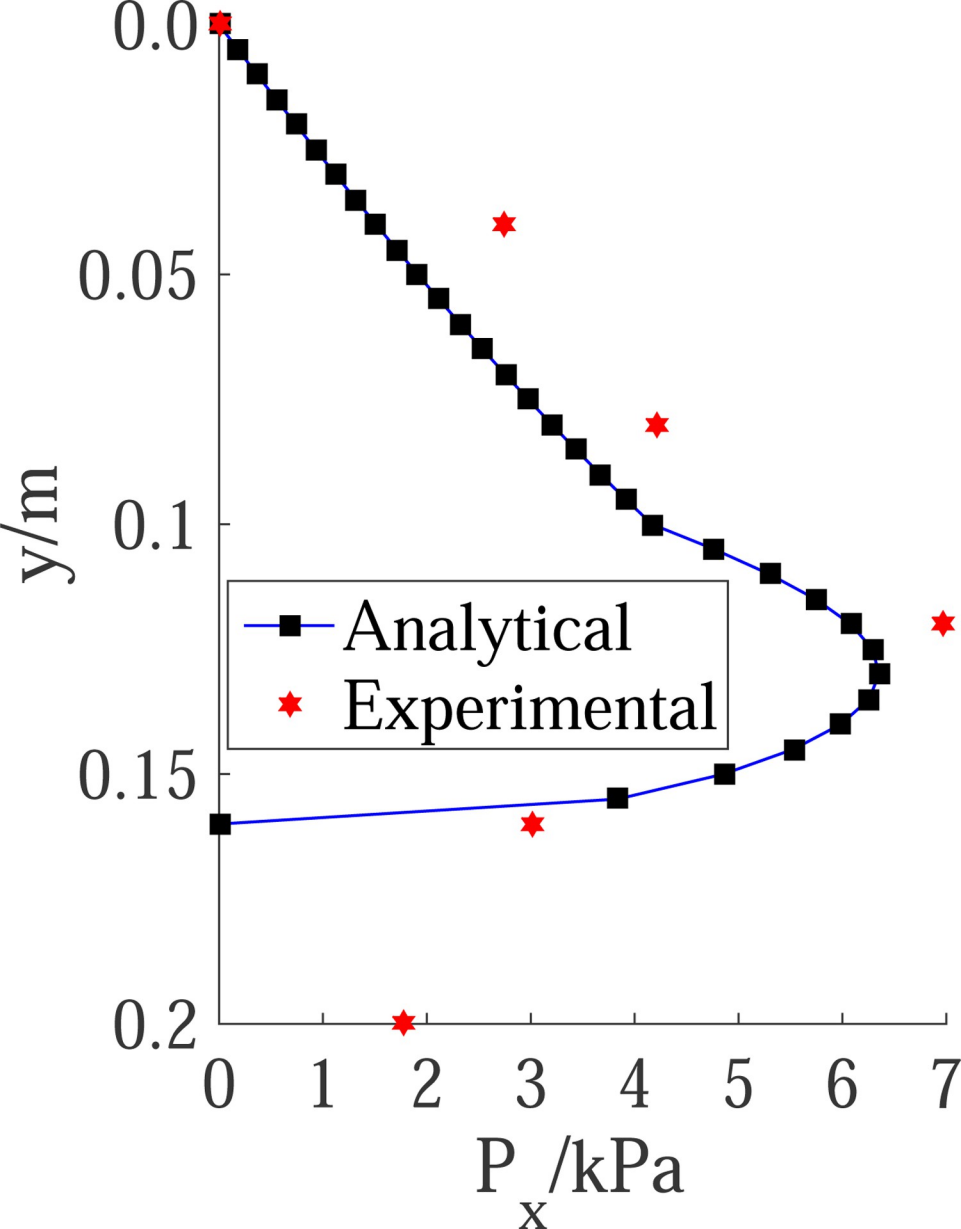
Comparison of passive pressure distribution with model test result.

The model test of the cantilever flexible retaining wall is carried out with 300mm high sand fill, and the final state is obtained. The corresponding horizontal displacement curve of the retaining wall is shown in Fig 18. From the test results in Fig 18, the displacement zero-point is at the height of 140mm, and the height of the passive zone is H = 160mm. The PIV test results are shown in Figs 7 and 9. The broken line sliding surface appears in the passive soil area, and the starting point of the sliding surface is located at the displacement zero-point. Taking the required horizontal displacement *S*p = H/10 = 16mm in the limit state, the height of the passive area fully entering the limit state is H_1_ = 100mm, and the height of the area not fully entering the limit state is H_2_ = 60mm. In line with the above geometric parameters, the calculation results obtained by the proposed method in this paper are compared with the earth pressure test results, as shown in Fig 19. The passive earth pressure at the displacement zero-point calculated by this method is close to zero. Thus, the calculated results are generally consistent with the earth pressure distribution in the test, and the solution is close to the measurement values. Therefore, the theoretical calculation method results are usually less than the test results, which is safe on the whole.

#### Comparison with other experimental studies

**Fig 20:**
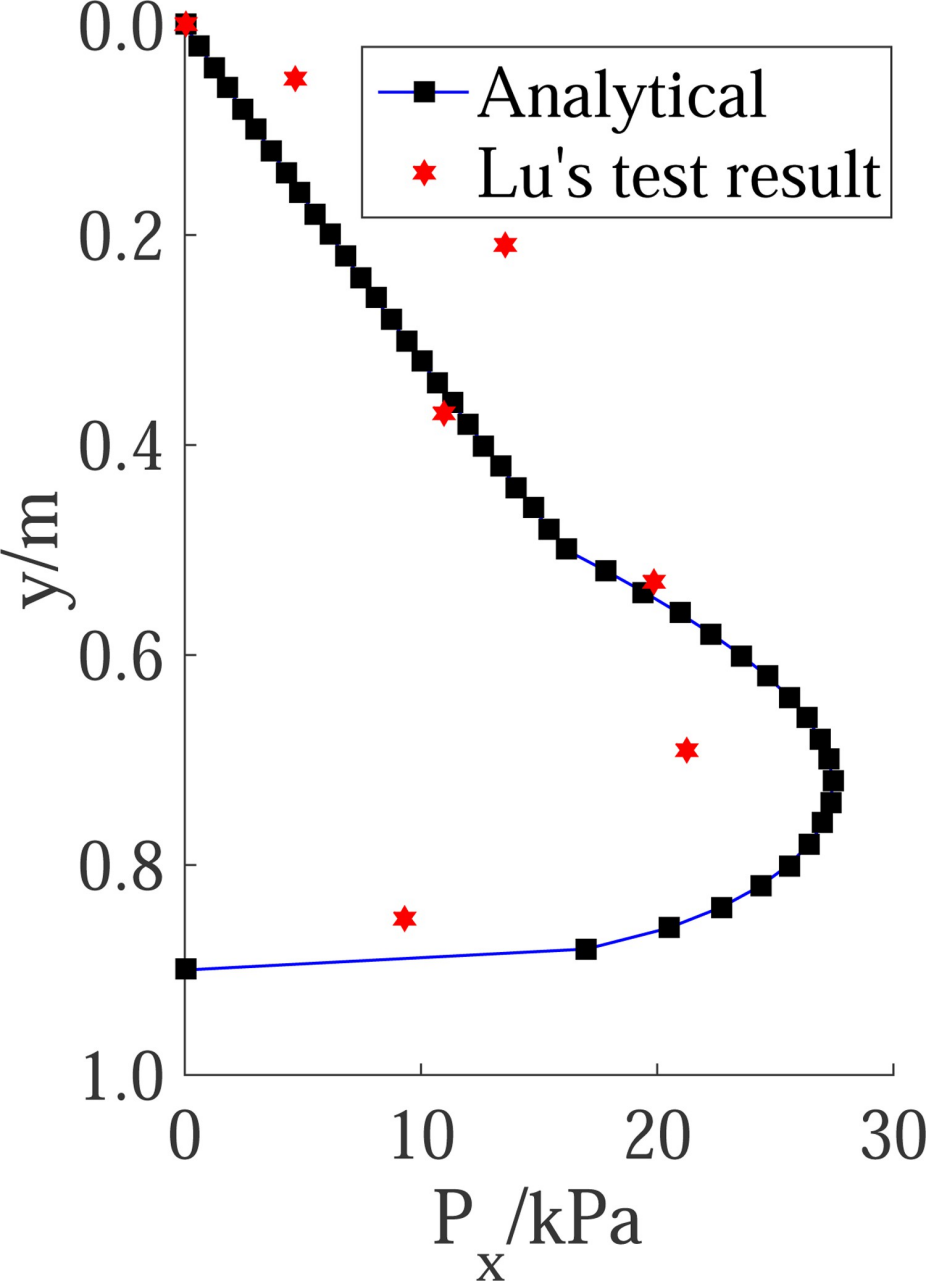
Comparison of passive pressure distribution with Lu’s result.

The results by the proposed method were compared with that of Lu’s test results [27], as shown in Fig 20. For dense sand, the height of the passive zone is H = 0.9m, and *S*p = 5H% is taken for the calculation. Based on the measured results of horizontal displacement in this paper, H_1_ = 0.5m, H_2_ = 0.4m. Thus, the distribution of passive earth pressure calculated is consistent with that measured in the test, and its value is also close.

## Conclusions

1. Under the flexural deformation mode of the cantilever flexible retaining wall in the foundation pit, the soil shear strain in the passive area increases continuously with the increase of flexural deformation and horizontal displacement, and an apparent shear fracture zone is formed at the final sliding failure. The soil fracture surface in the passive zone is a broken line fracture surface passing through the displacement zero-point and sliding out of the top surface of the soil.
2. The mode and magnitude of the displacement affect the mobilization of shear strength of the soil layer in the passive area. The displacement of the upper soil layer is large, reaching the passive limit state, and the strength parameters are taken as the maximum according to the limit state. On the other hand, the lower soil layer is in a non-limit state, and the strength parameters are taken nonlinearly according to the displacement.
3. The unit passive earth pressure in the limit state area increases continuously with the increase of depth. The unit passive earth pressure varies from nonlinear increase to rapid decrease after entering the non-limit state area.

## Supporting information

S1 Appendix (DOCX)

S2 Appendix (DOCX)
