# Supplementary material for: Non-limit passive earth pressure against cantilever flexible retaining wall in foundation pit considering the displacement
Source: PLoS One. 2022 Mar 11;17(3):e0264690. doi: 10.1371/journal.pone.0264690 (PMC8916651; doi:10.1371/journal.pone.0264690)
Supplement: S1 Appendix — (DOCX) [file pone.0264690.s001.docx]

**S1 Appendix**

The simplified equation can be obtained by omitting the high-order infinitesimal from the vertical force balance condition acting on the unit.

$\frac{dP_{y1}}{dy}=\gamma+\frac{\text{（}P_{y1}-N\text{）}\cot\beta_{1}+\tau_{1}+\tau_{2}}{H_{2}\cot\beta_{2}+\left(H_{1}-y\right)\cot\beta_{1}}$ （S1-1）

Combined formula (8) and formula (S1-1) can be obtained

$\frac{dP_{y1}}{dy}=\frac{\gamma}{1+b_{1}}+\frac{a_{2}P_{y1}}{b_{2}-y}$ （S1-2）

In which,$P_{x1}=k_{1}P_{y1}$, $\tau_{1}=P_{x1}\tan\delta$,

Where, *δ* is the friction angle between the wall and the soil, *φ* is the friction angle in the soil, and *k*_1_ is the passive lateral earth pressure coefficient. *k*_1_ can be expressed as

（S1-3）

which,

（S1-4）

$a_{2}=\frac{a_{1}}{1+b_{1}}$ （S1-5）

（S1-6）

$b_{2}=H_{2}\cot\beta_{2}\tan\beta_{1}+H_{1}$ （S1-7）

By using the boundary conditions, the vertical load on the bottom of the foundation pit is not calculated, that is, *P*_y1_=0 and *y*=0. Then the solution formula (S1-2) can be solved.

$P_{y1}=\frac{-\gamma(b_{2}-y)}{\left(1+b_{1}\right)(1+a_{2})}+\frac{\gamma b_{2}}{\left(1+b_{1}\right)(1+a_{2})}(\frac{b_{2}}{b_{2}-y})^{a_{2}}$ （S1-8）

Next, the unit lateral earth pressure can be obtained, *P*_x1_=*k*_1_*P*_y1_.When *y*=H_1_, the vertical stress *D* at the bottom of zone ① can be used as the loading condition at the top of zone ②, i.e

$D=P_{y1}\left|_{y=H_{1}}\right.=\frac{-\gamma(b_{2}-H_{1})}{\left(1+b_{1}\right)(1+a_{2})}+\frac{\gamma b_{2}}{\left(1+b_{1}\right)(1+a_{2})}(\frac{b_{2}}{b_{2}-H_{1}})^{a_{2}}$ (S1-9)
