# Supplementary material for: Non-limit passive earth pressure against cantilever flexible retaining wall in foundation pit considering the displacement
Source: PLoS One. 2022 Mar 11;17(3):e0264690. doi: 10.1371/journal.pone.0264690 (PMC8916651; doi:10.1371/journal.pone.0264690)
Supplement: S2 Appendix — (DOCX) [file pone.0264690.s002.docx]

**S2 Appendix**

Be considering the vertical force balance condition acting on the unit, and the simplified differential equation can be obtained by omitting the high-order infinitesimal,

$\frac{dP_{y2}}{dy}=\gamma+\frac{\text{（}P_{y2}-N\text{）}\cot\beta_{2}+\tau_{1}+\tau_{2}}{\left(H-y\right)\cot\beta_{2}}$ （S2-1）

Combining formula (9) and formula (S2-1), we can get

$\frac{dP_{y2}}{dy}=\frac{\gamma}{1-b_{3}}+\frac{cP_{y2}}{H-y}$ （S2-2）

in which,

$c=\frac{1-a_{3}+k_{2}\tan\delta_{m}\tan\beta_{2}}{1-b_{3}}$ （S2-3）

 （S2-4）

 （S2-5）

 （S2-6）

Using the loading condition on the top surface of zone ②, i.e. when *y*= H_1_, the vertical stress value *P*_y2_=*D*, the solution formula (S2-2) can be obtained

$P_{y2}=\frac{-\gamma(H-y)}{(\text{1-}b_{3})(\text{1+}c)}+[D+\frac{\gamma H_{2}}{(\text{1-}b_{3})(\text{1+}c)}](\frac{H-y}{H_{2}})^{-c}$ （S2-7）

Therefore, the lateral earth pressure of zone ② can be obtained, *P*_x2_=*k*_2_*P*_y2_
